# Cigarette smoking and prostate cancer aggressiveness among African and European American men

**DOI:** 10.1007/s10552-024-01883-3

**Published:** 2024-05-17

**Authors:** Edgar T. Ellis, Brian J. Fairman, Shelbie D. Stahr, Jeannette T. Bensen, James L. Mohler, Lixin Song, Eboneé N. Butler, L. Joseph Su, Ping-Ching Hsu

**Affiliations:** 1 Department of Environmental Health Sciences, Fay W. Boozman College of Public Health, University of Arkansas for Medical Sciences, 4301 W Markham St., #820, Little Rock, AR 72205-7190 USA; 2 Department of Epidemiology, Fay W. Boozman College of Public Health, University of Arkansas for Medical Sciences, Little Rock, AR 72205 USA; 3 Lineberger Comprehensive Cancer Center and Gillings School of Global Public Health, University of North Carolina at Chapel Hill, Chapel Hill, NC 27599 USA; 4 Department of Urology, Roswell Park Comprehensive Cancer Center, Buffalo, NY 14203 USA; 5 School of Nursing & Mays Cancer Center, University of Texas Health Science Center San Antonio, San Antonio, TX 78229 USA; 6 Department of Epidemiology, University of North Carolina at Chapel Hill, Chapel Hill, NC 27599 USA; 7 Peter O’Donnell Jr. School of Public Health, UT Southwestern Medical Center, Dallas, TX 75390 USA

**Keywords:** High-aggressive prostate cancer, Tobacco use, Racial disparities, Epidemiology

## Abstract

**Purpose:**

Smoking is a modifiable lifestyle factor that has not been established as a prostate cancer risk factor, nor emphasized in prostate cancer prevention. Studies have shown that African American (AA) smokers have a poorer cancer prognosis than European Americans (EAs), while having a lower prevalence of heavy smoking. We examined the relationship between cigarette smoking and prostate cancer aggressiveness and assessed racial differences in smoking habits on the probability of high-aggressive prostate cancer.

**Methods:**

Using data from the North Carolina-Louisiana Prostate Cancer Project (*n* = 1,279), prostate cancer aggressiveness was defined as high or low based on Gleason scores, serum prostate-specific antigen levels, and tumor stage. Cigarette smoking was categorized as current, former, or never smokers. Multivariable logistic regression was used to estimate adjusted odds ratios (OR) and 95% confidence intervals (CI).

**Results:**

Self-reported current (OR = 1.99; 95% CI 1.30–3.06) smoking was associated with high-aggressive prostate cancer relative to never smokers. When stratified by self-reported race, the odds of having high-aggressive cancer increased among AA current (OR = 3.58; 95% CI 2.04–6.28) and former smokers (OR = 2.21; 95% CI 1.38–3.53) compared to AA never smokers, but the odds were diminished among the EA stratum (*P*_self-reported race x smoking status_ = 0.003).

**Conclusion:**

Cigarette smoking is associated with prostate cancer aggressiveness, a relationship modulated by self-reported race. Future research is needed to investigate types of cigarettes smoked and metabolic differences that may be contributing to the racial disparities observed.

**Supplementary Information:**

The online version contains supplementary material available at 10.1007/s10552-024-01883-3.

## Introduction

Prostate cancer is the most diagnosed cancer in men in the United States (US) with an incidence rate of 109.9 per 100,000 men from 2015 to 2019 [1]. Classifications were developed to describe tumor aggressiveness, or how likely a tumor will spread from the prostate gland to other tissues or lead to metastatic disease [2]. The majority of prostate cancer cases are indolent, or slow growing, and will not cause harm during a man’s lifetime and do not require treatment [3]. About 15–30% of all cases are aggressive, in which radical treatment is necessary to avoid life-threatening metastatic spread [3]. The main risk factors for prostate cancer include age, race/ethnicity, family history, and genetic predisposition, all of which are non-modifiable [4–8]. Classifying the disease by aggressiveness, however, has led to researchers identifying modifiable lifestyle factors that may be associated with tumor invasion and metastasis [9]. One such modifiable lifestyle factor is cigarette smoking. Several meta-analyses and prospective cohort studies have found significant relationships linking cigarette smoking to later prostate cancer diagnosis and poorer survivability [9–16]. Several mechanisms have been hypothesized to moderate the relationship between cigarette smoking and prostate cancer aggressiveness, which include mutations in cancer progression genes, hormonal alterations, and enhanced tumor angiogenesis [17]. Nevertheless, a significant gap still exists in the literature about racial differences in prostate cancer aggressiveness and modifiable lifestyle factors such as cigarette smoking.

Racial disparities related to prostate cancer exist between European Americans and African Americans. African American men have higher prostate cancer incidence (176.2 vs. 103.5 per 100,000 men) and mortality (37.5 vs. 17.8 per 100,000 men) rates than European American men [1, 18–20]. Racial differences in smoking habits also exist between European and African American men [21–23]. Prior studies have shown that African American smokers have higher cotinine levels (the predominant nicotine metabolite) than European American smokers even when smoking fewer cigarettes per day [24–26], which may be due to higher nicotine intake per cigarette among African American smokers [24, 25, 27]. These studies highlight the fact that self-reported cigarettes per day predicts smoke intake more poorly in African American smokers than in European American smokers [27]. An additional factor is that approximately 85% of African American smokers use menthol cigarettes compared to 30% of European American smokers [28, 29]. Menthol provides a cooling sensory effect to reduce the harshness of cigarette smoke and has been shown to modify smoking behavior by increasing puff volumes and exposure to hazardous chemicals [21–23]. These differences could be associated with the racial disparities present in prostate cancer diagnosis and tumor aggressiveness [21–23].

This study aimed to examine the relationship between cigarette smoking and prostate cancer aggressiveness, and to assess the racial differences in smoking habits on the probability of high-aggressive cancer. The hypothesis was that current smoking status will be associated with increased odds of high-aggressive prostate cancer diagnosis compared to former/non-smoking status. Furthermore, we hypothesized that the relationship between cigarette smoking and prostate aggressiveness will be modulated by self-reported race due to different smoking habits.

## Methods

### Study population

This cross-sectional study used data from the North Carolina–Louisiana Prostate Cancer Project (PCaP). PCaP is a population-based cohort of incident prostate cancer cases from North Carolina (NC) and Louisiana (LA) collected from 2004 to 2009 designed to examine prostate cancer populations from two states with variable prostate cancer incidence and study the racial disparities associated with the disease. Eligible PCaP participants included those aged 40–79 years old at diagnosis, were physically and mentally able to complete the interview, could complete the interview in English, and self-identified as at least part Black/African American or Caucasian/European American/White. Participants who indicated more than one racial group were asked if one best describes them, and if not, multiple groups were recorded. The present study reports Black/African American participants as “African American,” and Caucasian/European American/White participants as “European American”. Comparable participant ascertainment and enrollment rates by race and state/study site throughout the enrollment period was accomplished using a randomized recruitment procedure. Participants were identified by rapid case ascertainment through state cancer registries and diagnosing physicians were contacted for permission to contact the patient, and if permission was obtained patients were contacted by study staff to confirm eligibility and schedule an interview. The time from diagnosis to survey completion varied between 1 and 3 months. Complete PCaP methodology can be found elsewhere [30].

### Measures

The main exposure of interest in this study was self-reported smoking status defined as current, former, or never smoker. Participants completed survey questions at PCaP enrollment on whether they had smoked at least 100 cigarettes or 5 packs in their lifetime and to recall whether they were smoking at the time of prostate cancer diagnosis. Current smokers were those who smoked ≥ 100 cigarettes or ≥ 5 packs in their lifetime and responded “yes” to smoking cigarettes at the time of prostate cancer diagnosis. Former smokers were those who smoked ≥ 100 cigarettes or ≥ 5 packs in their lifetime but responded “no” to smoking cigarettes at the time of prostate cancer diagnosis. Never smokers were those who smoked < 100 cigarettes or < 5 packs in their lifetime or had never smoked a cigarette. Further analyses were performed categorizing smoking status into 1) never, former (> 23 years since cessation), former (≤ 23 years since cessation), or current smokers, and 2) never smoker or former/current (ever) smoker combined categorized by total pack-years smoked in tertiles. The cutoff for years since cessation were based on the median value for former smoker low-aggressive cases. Tertile cutoffs at the 33.33 and 66.67 percentages were based on the pack-year values for ever-smoker low-aggressive cases. Pack-years were calculated by multiplying the total number of years smoked by the average number of cigarette packs smoked per day (20 cigarettes per pack).

The outcome of interest in this study was prostate cancer aggressiveness at diagnosis defined as high-aggressive or low-aggressive. Medical records were requested from the diagnosing physicians of consenting research participants. Abstractors derived clinical stage according to a standardized protocol, and staff used a relational database to abstract information regarding prostate cancer screening examinations and laboratory assays, imaging examinations used in staging, clinical stage and grade, and initial treatment information. High-aggressive prostate cancer was defined as a case with Gleason score (sum of two Gleason grades from two areas that make up most of the cancer) greater than or equal to 8, or diagnostic serum prostate-specific antigen (PSA) greater than 20 ng/mL, or Gleason score 7 and stage T3–T4. Low-aggressive prostate cancer was defined as a case with Gleason score less than 7 and stage T1–T2 and PSA < 10 ng/mL. Intermediately aggressive cases were excluded from the analysis to avoid potential misclassification given limitations in the Gleason scoring system [31, 32] and differing classification systems between organizations during PCaP recruitment [33]. The focus of this study was the investigation of racial differences in smoking habits between the two extreme phenotypes of prostate cancer that may or may not cause considerable harm in a man’s lifetime.

Confounders were selected a priori based on previous literature and known risk factors for prostate cancer aggressiveness. Covariates included age (continuous), self-reported race (European American or African American), family history of prostate cancer (no/unknown affected first degree relative, at least one affected first degree relative), previous screening history (no previous PSA or digital rectal exam (DRE), PSA or DRE but not both, or PSA and DRE), education (less than high school, high school graduate, some college/vocational, or college degree), income (less than $30,000, $30,001–$70,000, or greater than $70,001), body mass index (BMI) (normal/underweight (< 25 kg/m^2^), overweight (25–29.9 kg/m^2^), or obese (30 + kg/m^2^)), alcohol consumption (yes or not currently), health insurance status within a year of diagnosis (yes or no), PCaP study site (University of North Carolina at Chapel Hill (UNC) or Louisiana State University Health Sciences Center (LSUHSC)), and other tobacco use (yes or no). Other tobacco use was defined as ever using pipes, cigars, cigarillos, chewing tobacco, or snuff.

### Statistical analysis

Participant demographic and clinical characteristics of the final analytic sample were calculated and stratified according to prostate cancer aggressiveness and by self-reported race. Unadjusted and adjusted multivariable unconditional logistic regression models were used to estimate the odds ratios (OR) and 95% confidence intervals (CI) to describe the association between self-reported smoking status and prostate cancer aggressiveness. Never smokers were used as the reference group for all regression models. To assess for confounding, multivariable logistic regression models were used to determine the association of covariates with the exposure and outcome, the magnitude and direction of stratified outcomes compared to the unadjusted outcome, and the percent difference between unadjusted and adjusted models with covariates. Covariates that did not change the OR by > 10% were dropped from the model. Interaction was assessed by the heterogeneity of effects across strata for multiplicative interaction and by the joint effects model for additive interaction with a cross product term included in the regression model [34]. Chi-square tests for categorical significance and T-tests for continuous significance were used for descriptive analysis. A 2-tailed *p*-value < 0.05 was used to indicate statistical significance across all analyses. Statistical analysis was conducted using SAS, version 9.4 (SAS Institute, Cary, NC).

## Results

### Study participant characteristics

A total of 2,258 eligible participants aged 40–79 years were enrolled in PCaP. Participants were excluded from analysis if they had insufficient data to classify prostate cancer aggressiveness (*n* = 85), were classified as intermediate prostate cancer aggressiveness (*n* = 676), had missing/refused answers for smoking status (*n* = 3), health insurance (*n* = 8), education (*n* = 1), BMI (*n* = 16), previous screening history (*n* = 18), income (*n* = 123), pack-years smoked (*n* = 46), and years smoked (*n* = 1). Two participants were excluded due to extremely high cigarettes per day (*n* = 1) and erroneously entered total years smoked (*n* = 1). The final analytic sample included 1,279 participants with complete covariate information. Table 1 presents the demographic characteristics of the study participants by prostate cancer aggressiveness and self-reported race. Compared to low-aggressive prostate cancer cases (*n* = 904), a higher proportion of high-aggressive prostate cancer cases (*n* = 317) were current smokers (21.8% vs. 13.0%), were African American (56.7% vs. 43.9%), had no previous screening history (20.3% vs. 8.1%), did not have health insurance (15.2% vs. 7.9%), had a college degree (21.2% vs. 16.2%), and had an income of less than $30,000 (49.7% vs. 29.5%). High-aggressive cases were older on average, had a higher average BMI, and had more average total years smoked than low-aggressive cases.

**Table 1:** Study participant demographic, smoking^a^, and clinical characteristics by prostate cancer aggressiveness at diagnosis^b^ and self-reported race

Characteristic	All cases	High-aggressive		Low-Aggressive	
Self-reported race	Total	African American	European American	African American	European American
	(*n* = 1,279)	(*n* = 187)	(*n* = 143)	(*n* = 417)	(*n* = 532)
	Mean (SD)				
Age at diagnosis, y	62.7 (7.9)	61.2 (7.8)	66.4 (7.4)	62.4 (7.7)	63.2 (7.8)
Body mass index, kg/m^2^	29.2 (5.5)	29.6 (6.8)	30.6 (5.2)	29.0 (5.5)	28.9 (4.9)
Total years smoked^c^, y	29.2 (15.5)	33.0 (15.2)	27.9 (16.1)	30.6 (14.6)	26.5 (15.7)
Years since cessation^d^, y	22.4 (14.3)	18.3 (14.1)	27.1 (16.6)	18.9 (12.7)	24.8 (13.8)
	Median (IQR)				
Cigarettes per day^c^, no	20.0 (10.0)	10.0 (13.0)	20.0 (20.0)	14.0 (13.0)	20.0 (10.0)
Pack-years smoked^c^	23.0 (35.0)	19.0 (31.0)	34.0 (53.0)	20.0 (31.5)	27.0 (38.0)
Prostate specific antigen (ng/mL)	5.3 (3.5)	21.8 (28.8)	10.3 (20.6)	5.1 (2.4)	4.6 (2.3)
Missing*	24	12	10	1	1
	Number (%)				
Smoking status					
Never	453 (35.4)	34 (18.2)	54 (37.8)	155 (37.2)	210 (39.5)
Former	631 (49.3)	95 (50.8)	75 (52.5)	187 (44.8)	274 (51.5)
Current	195 (15.3)	58 (31.0)	14 (9.8)	75 (18.0)	48 (9.0)
Screening history					
PSA and DRE	880 (68.8)	77 (41.2)	103 (72.0)	271 (65.0)	429 (80.6)
PSA or DRE	255 (19.9)	55 (29.4)	28 (19.6)	90 (21.6)	82 (15.4)
No previous screening history	144 (11.3)	55 (29.4)	12 (8.4)	56 (13.4)	21 (4.0)
Family history of prostate cancer					
No/Unknown	961 (75.1)	138 (73.8)	115 (80.4)	313 (75.1)	395 (74.3)
Yes	318 (24.9)	49 (26.2)	28 (19.6)	104 (24.9)	137 (25.8)
Health insurance status					
Yes	1154 (90.2)	144 (77.0)	136 (95.1)	363 (87.1)	511 (96.1)
No	125 (9.8)	43 (23.0)	7 (4.9)	54 (13.0)	21 (4.0)
Education status					
College degree	385 (30.1)	19 (10.2)	51 (35.7)	75 (18.0)	240 (45.1)
Some college/vocational	331 (25.9)	50 (26.7)	45 (31.5)	111 (26.6)	125 (23.5)
High school graduate	316 (24.7)	48 (25.7)	24 (16.8)	119 (28.5)	125 (23.5)
Less than high school	247 (19.3)	70 (37.4)	23 (16.1)	112 (26.9)	42 (7.9)
Income					
$70,001 or greater	381 (29.8)	20 (10.7)	52 (36.4)	79 (18.9)	230 (43.2)
$30,001–$70,000	454 (35.5)	42 (22.5)	52 (36.4)	161 (38.6)	199 (37.4)
Less than $30,000	444 (34.7)	125 (66.8)	39 (27.3)	177 (42.5)	103 (19.4)
Study site of enrollment					
UNC	627 (49.0)	87 (46.5)	65 (45.5)	200 (48.0)	275 (51.7)
LSUHSC	652 (51.0)	100 (53.5)	78 (54.6)	217 (52.0)	257 (48.3)
Alcohol use					
No	495 (38.7)	86 (46.0)	43 (30.1)	186 (44.6)	180 (33.8)
Yes	784 (61.3)	101 (54.0)	100 (69.9)	231 (55.4)	352 (66.2)
Other tobacco use					
No	997 (78.0)	152 (81.3)	105 (73.4)	347 (83.2)	393 (73.9)
Yes	282 (22.0)	35 (18.7)	38 (26.6)	70 (16.8)	139 (26.1)
Gleason sum					
< 7	979 (76.6)	16 (8.6)	14 (9.8)	417 (100.0)	532 (100.0)
7^e^	75 (5.9)	50 (26.9)	25 (17.5)	–	–
≥ 8	224 (17.5)	120 (64.5)	104 (72.7)	–	–
Missing**	1	1	–	–	–
Tumor stage					
T1–T2	1232 (97.7)	161 (91.5)	122 (89.7)	417 (100.0)	532 (100.0)
T3–T4	29 (3.3)	15 (8.5)	14 (10.3)	–	–
Missing***	18	11	7	–	–

*PSA* Prostate Specific Antigen, *DRE* Digital Rectal Examination, *UNC* University of North Carolina at Chapel Hill; LSUHSC, Louisiana State University Health Sciences Center

^a^Smoking status defined by survey questions on whether participants had smoked more than 100 cigarettes (5 packs) in their lifetime, and if they were currently smoking at time of prostate cancer diagnosis. Current (> 100 cigarettes and currently smoking), former (> 100 cigarettes and not currently smoking), never (< 100 cigarettes)

^b^Prostate cancer aggressiveness defined by Gleason sum, tumor stage, and PSA level at diagnosis. High-aggressive (Gleason sum ≥ 8 or PSA > 20 ng/mL or Gleason sum = 7 and tumor stage 3–4), low-aggressive (Gleason sum < 7 and tumor stage 1–2 and PSA < 10 ng/mL)

^c^Current and former smokers only

^d^Former smokers only

^e^Gleason sum = 7 includes 3 + 4 and 4 + 3

*Missing PSA level high-aggressive cases were all Gleason sum ≥ 8, and low-aggressive cases were Gleason sum < 7 and tumor stage 1

**Missing Gleason sum high-aggressive case had PSA > 20 ng/mL

***Missing tumor stage cases were either Gleason sum ≥ 8 or PSA > 20 ng/mL

Compared to European Americans (*n* = 675), a higher proportion of African Americans (*n* = 604) were current smokers (22.0% vs. 9.2%), had no previous screening history (18.4% vs. 4.9%), had no health insurance (16.1% vs. 4.2%), had a less than high school education (30.1% vs. 9.6%), had an income of less than $30,000 (50.0% vs. 21.0%), did not currently drink alcohol (45.0% vs. 33.0%), and did not smoke other tobacco (82.6% vs. 73.8%). African Americans were younger on average (Mean (SD) = 61.5 (7.8) vs. 63.8 (7.8)), had greater total years smoked (Mean (SD) = 31.5 (14.8) vs. 26.8 (15.7)), less time since cessation (Mean (SD) = 18.7 (13.1) vs. 25.3 (14.5)), less cigarettes smoked per day (Median (IQR) = 12.0 (13.0) vs. 20.0 (20.0)), and less total pack-years smoked (Median (IQR) = 19.0 (31.1) vs. 28.5 (40.0)) than European Americans. Clinical characteristics between racial groups were significantly different for Gleason sum and PSA level, with European American men having a higher proportion of low (< 7) Gleason sum (80.9% vs. 71.8%), and African American men having a higher median PSA (Median (IQR) = 5.72 (4.58) vs. 4.87 (2.93)).

### Association between self-reported smoking status and prostate cancer aggressiveness

The unadjusted and adjusted odds ratios and 95% confidence intervals for self-reported smoking statuses are provided in Table 2. Current, but not former, smokers had significantly increased odds of having a high-aggressive prostate cancer diagnosis than never smokers after controlling for confounders (OR = 1.99; 95% CI 1.30–3.06; *P*_trend_ = 0.001). Splitting former smokers by the low-aggressive case years since cessation median, neither former smoker categories were significantly associated with high-aggressive prostate cancer, but there was a significant trend across categories (*P*_trend_ = 0.003). Categorizing ever-smokers by tertiles of pack-years smoked in the low-aggressive cases, tertile 2 (12.3 – 37 pack-years) produced significantly higher odds of association with high-aggressive prostate cancer compared to never smokers (OR = 1.58; 95% CI 1.10–2.28). While tertile 1 and 3 did not produce significant results, there was still a significant trend across categories (*P*_trend_ = 0.03).

**Table 2:** Unadjusted and adjusted^a^ odds ratios and 95% confidence intervals for the association between self-reported smoking status^b^ and high-aggressive prostate cancer^c^ at diagnosis

	High-/Low-aggressive cases	Unadjusted		Adjusted	
		OR	95% CI	OR	95% CI
Smoking status					
Never	88/365	1.0	(ref.)	1.0	(ref.)
Former	170/461	1.53	1.14–2.05	1.33	0.98–1.81
Current	72/123	2.43	1.67–3.52	1.99	1.30–3.06
		*P*_trend_ ≤ 0.001		*P*_trend_ = 0.001	
Smoking status with years since cessation^d^					
Never	88/365	1.0	(ref.)	1.0	(ref.)
Former > 23 years since quit	79/222	1.48	1.04–2.09	1.36	0.94–1.96
Former ≤ 23 years since quit	91/239	1.58	1.13–2.21	1.30	0.91–1.86
Current	72/123	2.43	1.67–3.52	1.99	1.29–3.05
		*P*_trend_ ≤ 0.001		*P*_trend_ = 0.004	
Pack-years smoked^e^					
Never	88/365	1.0	(ref.)	1.0	(ref.)
Tertile 1	72/195	1.53	1.07–2.19	1.35	0.93–1.97
Tertile 2	87/195	1.85	1.31–2.61	1.58	1.10–2.28
Tertile 3	83/194	1.78	1.26–2.51	1.42	0.97–2.07
		*P*_trend_ ≤ 0.001		*P*_trend_ = 0.03	

^a^Adjusted for age, self-reported race, family history, screening history, body mass index, health insurance, education, and income

^b^Smoking status defined by survey questions on whether participants had smoked more than 100 cigarettes (5 packs) in their lifetime, and if they were currently smoking at time of prostate cancer diagnosis. Current (> 100 cigarettes and currently smoking), former (> 100 cigarettes and not currently smoking), never (< 100 cigarettes)

^c^Prostate cancer aggressiveness defined by Gleason sum, tumor stage, and PSA level at diagnosis. High-aggressive (Gleason sum ≥ 8 or PSA > 20 ng/mL or Gleason sum = 7 and tumor stage 3–4), low-aggressive (Gleason sum < 7 and tumor stage 1–2 and PSA < 10 ng/mL)

^d^Former smoker cutoff based on low-aggressive case years since cessation median

^e^Tertile cutoffs based on low-aggressive former and current smoker’s total pack-years smoked (number of cigarette packs per day x number of total years smoked) (T1 ≤ 12.3; 12.3 < T2 ≤ 37; T3 > 37 pack-years)

To assess for interactions, the heterogeneity of effects (Table 3) and joint effects (Table 4) models were used adjusting for confounders and including a cross-product term in the regression model. There was significant positive multiplicative interaction between smoking status and self-reported race when including a cross-product term in the regression model (*β*_*current smoker x African American*_ = 1.32; *p* = 0.003) (*β*_*former smoker x African American*_ = 0.94; *p* = 0.003). Within strata, African American current (OR = 3.58; 95% CI 2.04–6.28) and former (OR = 2.21; 95% CI 1.38–3.53) smokers had significantly increased odds of high-aggressive cancer compared to African American never smokers. In the European American stratum, the ORs for current and former smokers were not significantly associated with high-aggressive prostate cancer. Dichotomizing age into older and younger groups based on the average age of low-aggressive cases (62.3 years), there was no significant multiplicative interaction between age and smoking on high-aggressive prostate cancer (*p* = 0.10). Tests for interaction between smoking and previous screening history did not show evidence for significant multiplicative interaction (*p* = 0.59). Heterogeneity of effects using the alternative exposure classifications with self-reported race (dichotomized former smokers and total pack-year tertiles) produced similar results to the main exposure (Supplemental Tables 1 & 2).

**Table 3:** Adjusted^a^ multivariable logistic regression associations between self-reported smoking status^b^ and high-aggressive prostate cancer^c^ stratified by self-reported race, age^d^, and screening history

	Smoking status	High-/Low-aggressive cases	OR	95% CI
Main effect	Never	88/365	1.00	(ref.)
	Former	170/461	1.36	1.00–1.86
	Current	72/123	1.97	1.29–3.03
Self-reported race				
African American	Never	34/155	1.00	(ref.)
	Former	95/187	2.21	1.38–3.53
	Current	58/75	3.58	2.04–6.28
European American	Never	54/210	1.00	(ref.)
	Former	75/274	0.87	0.57–1.31
	Current	14/48	0.96	0.47–1.95
	*P* (multiplicative interaction by race) = 0.003			
Age				
Younger (≤ 62.3)	Never	36/212	1.00	(ref.)
	Former	52/186	1.50	0.92–2.43
	Current	51/78	2.69	1.55–4.66
Older (> 62.3)	Never	52/153	1.00	(ref.)
	Former	118/275	1.26	0.85–1.88
	Current	21/45	1.12	0.59–2.15
	*P* (multiplicative interaction by age) = 0.10			
Screening history				
No previous screening	Never	15/23	1.00	(ref.)
	Former	26/25	1.50	0.61–3.67
	Current	26/29	1.63	0.66–4.00
PSA or DRE	Never	18/63	1.00	(ref.)
	Former	40/80	1.35	0.69–2.65
	Current	25/29	3.16	1.43–6.96
PSA and DRE	Never	55/279	1.00	(ref.)
	Former	104/356	1.30	0.89–1.90
	Current	21/65	1.70	0.93–3.11
	*P* (multiplicative interaction by previous screening history) = 0.59			

^a^Adjusted for age (in age-unstratified model), self-reported race (in race-unstratified model), family history, screening history, body mass index, health insurance, education, and income

^b^Smoking status defined by survey questions on whether participants had smoked more than 100 cigarettes (5 packs) in their lifetime, and if they were currently smoking at time of prostate cancer diagnosis. Current (> 100 cigarettes and currently smoking), former (> 100 cigarettes and not currently smoking), never (< 100 cigarettes)

^c^Prostate cancer aggressiveness defined by Gleason sum, tumor stage, and PSA level at diagnosis. High-aggressive (Gleason sum ≥ 8 or PSA > 20 ng/mL or Gleason sum = 7 and tumor stage 3–4), low-aggressive (Gleason sum < 7 and tumor stage 1–2 and PSA < 10 ng/mL)

^d^Age dichotomized based on the average age of low-aggressive cases

**Table 4:** Joint effects^a^ of self-reported smoking status^b^ and self-reported race associated with high-aggressive prostate cancer^c^ at diagnosis

Smoking Status	European American			African American		
	High-/Low-aggressive	OR	95% CI	High-/Low-aggressive	OR	95% CI
Never	54/210	1.0	(ref.)	34/155	0.65	0.39–1.09
Former	75/274	0.87	0.57–1.31	95/187	1.44	0.94–2.21
Current	14/48	0.96	0.47–1.95	58/75	2.33	1.35–4.04

^a^Multivariable logistic regression model adjusted for age, family history, screening history, body mass index, health insurance, education, and income

^b^Smoking status defined by survey questions on whether participants had smoked more than 100 cigarettes (5 packs) in their lifetime, and if they were currently smoking at time of prostate cancer diagnosis. Current (> 100 cigarettes and currently smoking), former (> 100 cigarettes and not currently smoking), never (< 100 cigarettes)

^c^Prostate cancer aggressiveness defined by Gleason sum, tumor stage, and PSA level at diagnosis. High-aggressive (Gleason sum ≥ 8 or PSA > 20 ng/mL or Gleason sum = 7 and tumor stage 3–4), low-aggressive (Gleason sum < 7 and tumor stage 1–2 and PSA < 10 ng/mL)

Further assessing interaction between self-reported smoking and race in the joint effects model with European American never smokers as a common reference group (Table 4), African American current smokers (OR = 2.23; 95% CI 1.35–4.04) had significantly increased odds of having high-aggressive prostate cancer. But, African American former and never smokers and European American current and former smokers had null/inverse associations with high-aggressive prostate cancer compared to the reference group. To explore for measures of additive interaction with inverse associations in the joint effects model, the coding scheme for race was reversed so that African American never smokers were the reference group [34–36]. Comparing current/never smokers between racial groups with African American never smokers as the reference group, the relative excess risk due to interaction (RERI) was statistically significant in the negative direction (RERI = − 2.82; 95% CI − 5.22 – − 0.42) [34].

## Discussion

This study evaluated whether self-reported cigarette smoking status at the time of diagnosis was associated with high-aggressive prostate cancer and if self-reported race modulated this association. The results indicate that current, but not former, smoking increases the odds of having a high-aggressive prostate cancer diagnosis, and that African American smokers have significantly increased odds of aggressive disease compared to European American smokers. This study is among the few to address the literature gap pertaining to racial differences in smoking habits and the association with aggressive prostate cancer diagnosis.

Previous studies examining cigarette smoking and prostate cancer aggressiveness have generally agreed that cigarette smoking is associated with aggressive disease [17], rather than the incidence of cancer itself, and that cigarette smoking increases risk of biochemical recurrence [16, 37], and mortality [15, 37]. The observed results support the argument that currently smoking at the time of diagnosis is associated with high-aggressive disease defined by three measurements (Gleason sum, PSA score, or tumor stage). In a population-based case–control study, investigators found that current smokers and those with > 40 pack-years were at significantly increased risk of having aggressive forms of the disease [38]. While there was a significant trend in pack-year tertiles in the current study, the highest tertile smokers did not have significantly increased odds of a high-aggressive diagnosis, contrary to the previous work [38]. The current study also found no significant relationship between smoking cessation and high-aggressive disease among former smokers, whereas the previous study found inverse, though insignificant, associations [38]. The findings of current smokers having increased odds of high-aggressive disease persist after adjusting for confounders such as previous screening history, which is thought to confound the association because cigarette smokers may be less health conscious than non-smokers and receive screenings less often [14]. The most significant finding from this work, however, is that racial differences in smoking habits positively modulate the association between cigarette smoking and prostate cancer aggressiveness, which few previous works have investigated due to small sample sizes and/or recruitment of African American men.

This study is one of the few to have investigated racial disparities with smoking habits in relation to high-aggressive prostate cancer at diagnosis, which can lead to worse survivability after treatment and recurrence, especially in African American men [18, 20, 23, 39]. African American smokers were significantly more likely to be diagnosed with high-aggressive disease compared to European Americans, even though African American smokers had less cigarettes smoked per day and less pack-years smoked. A previous case–control study investigated the racial differences between African American and European American men in relation to cigarette smoking and high-grade prostate cancer [40]. The authors found that, as in the present study, African American men had a higher prevalence of smoking and less rates of heavy smoking, while also having higher odds for high-grade cancer [40]. The present study builds upon limitations discussed in Murphy et al. [40] by increasing the European American sample size, and findings within this group are also consistent with European American smokers not having significantly increased odds of high-aggressive cancer. The racial disparities observed within the African American group may imply that African American smokers smoke more intensely (e.g. puff volume) and/or that these men metabolize cigarettes differently than European American smokers.

One hypothesis that may explain the relative decrease in smoking habits (cigarette per day, years smoked, and pack-years smoked) but increase in aggressive disease among racial groups could be mentholated cigarette use in the African American population [29, 41]. Menthol provides a cooling sensory effect to reduce the harshness of cigarette smoke and has been hypothesized to modify smoking behavior by increasing the puff volumes that leads to increased exposure to hazardous chemicals [28, 41, 42]. It has been shown in previous studies that African American men smoke fewer cigarettes per day, which could be attributed to smoking mentholated cigarettes [28]. Benowitz et al. conducted a correlation study for cigarettes per day and biomarkers of nicotine and carcinogen exposure in African and European American smokers and found significant differences between racial groups and the predictive power of smoke intake [27]. On average, African Americans smoke fewer cigarettes per day than European Americans [43], but even when selection criteria makes these values similar between groups, African Americans have a flat relationship between cigarettes per day and nicotine or carcinogen exposure [27]. The authors discuss that smoking patterns seen in African American smokers may be due to intensive smoking, in which less cigarettes are smoked per day, but a greater intake of smoke constituents is observed [27].

Metabolic differences could also explain the findings of racial differences between smokers. A clinical investigation by Perez-Stable et al. found that African American smokers had higher cotinine levels per cigarette smoked compared to European American smokers, which may point to mutagenic and non-mutagenic differences in metabolism among racial groups [25, 44, 45]. Mentholated cigarette smoking may also contribute to metabolic differences between racial groups, since menthol can inhibit CYP2A6 enzyme activity, a main pathway for nicotine metabolism [46–48]. The types of cigarettes smoked and metabolic activity are unmeasured in the current study, but the descriptive and analytical results observed on the smoking habits of African American men in relation to cancer aggressiveness point to the need for further investigation.

### Strengths and limitations

Strengths of this study included well-defined prostate cancer diagnosis and aggressiveness classifications, which were thoroughly abstracted to avoid mistakes in data collection/upkeep. Another strength of this study is derived from the PCaP methodology, which controlled for both investigator and subject selection bias due to the randomized recruitment procedure during the enrollment period, and due to all participants having an incident prostate cancer diagnosis rather than having prevalent prostate cancer or recurrence. Another strength was the study population, which was representative of both North Carolina and Louisiana prostate cancer case target populations in terms of race and diagnosis.

A limitation of the present study was the survey questions on cumulative smoking status, which may not give a complete picture of the smoking differences and smoking variations in sub-groups over a lifetime [44, 49]. A significant limitation was the lack of information on types of cigarettes smoked (e.g. mentholated cigarettes), smoking intensity/duration (e.g. puff volume), and metabolite concentrations (e.g. cotinine and 3-hydroxy-cotinine) to address racial differences in smoking behaviors and metabolism of cigarette smoking [25, 27]. There was potential for recall bias regarding self-reported cigarette use if smokers were more or less likely to recall their smoking habits accurately at the time of prostate cancer diagnosis, in which case the direction of bias may move away from the null if one outcome group differentially misclassified their exposure status [37, 49]. Residual confounding due to unmeasured characteristics cannot be completely ruled out such as the intensity of smoking or smoking measured through the life-course, or other measures such as cotinine metabolism or types of cigarettes smoked. The cross-sectional study design was limited in its ability to address temporality in this association, and prospective studies using life-course approaches would offer a better design to investigate the association between smoking and prostate cancer aggressiveness [49].

## Conclusion

This case-only cross-sectional study found a significant increase in the odds of current smokers being diagnosed with high-aggressive prostate cancer, an association that is modulated by self-reported race. The findings are consistent with previous literature that African American men have a higher prevalence of smoking, while having a lower prevalence of heavy smoking based on cigarettes smoked per day and pack-years smoked compared to European Americans, but have higher odds of high-aggressive disease. Future studies are needed to examine the gaps in knowledge regarding whether the type of cigarette smoked and metabolic differences are contributing to the high-aggressive prostate cancer racial disparities observed in the present study population.

## Supplementary Information

Below is the link to the electronic supplementary material.

Supplementary file1 (DOCX 20 KB)

## Data Availability

The data underlying this article cannot be shared due to the privacy of individuals that participated in the study. Summary level data will be shared on request to the corresponding author with permission of the advisory committee from the North Carolina–Louisiana Prostate Cancer Project.
