# Supplementary material for: Cigarette smoking and prostate cancer aggressiveness among African and European American men
Source: Cancer Causes Control. 2024 May 17;35(9):1259–69. doi: 10.1007/s10552-024-01883-3 (PMC11377453; doi:10.1007/s10552-024-01883-3)
Supplement: Supplementary file 1 — Supplementary file1 (DOCX 20 KB) [file 10552_2024_1883_MOESM1_ESM.docx]

**Cigarette Smoking and Prostate Cancer Aggressiveness Among African and European American Men**

^1^Edgar T. Ellis, ^2^Brian J. Fairman, ^1^Shelbie D. Stahr, ^3^Jeannette T. Bensen, ^4^James L. Mohler, ^5^Lixin Song, ^6^Eboneé N. Butler, ^7^L. Joseph Su, ^1#^Ping-Ching Hsu

^1^Department of Environmental Health Sciences, Fay W. Boozman College of Public Health, University of Arkansas for Medical Sciences, Little Rock, AR 72205, USA;

^2^Department of Epidemiology, Fay W. Boozman College of Public Health, University of Arkansas for Medical Sciences, Little Rock, AR 72205, USA;

^3^Lineberger Comprehensive Cancer Center and Gillings School of Global Public Health, University of North Carolina at Chapel Hill, Chapel Hill, NC 27599, USA;

^4^Department of Urology, Roswell Park Comprehensive Cancer Center, Buffalo, NY 14203, USA;

^5^School of Nursing & Mays Cancer Center, University of Texas Health Science Center San Antonio, San Antonio, TX 78229, USA;

^6^Department of Epidemiology, University of North Carolina at Chapel Hill, Chapel Hill, NC 27599, USA;

^7^Peter O’Donnell Jr. School of Public Health, UT Southwestern Medical Center, Dallas, Texas 75390, USA

**Word Count**: 3,887

**^#^Corresponding Author**:

Ping-Ching Hsu, PhD, MSc.

Associate Professor

Department of Environmental Health Sciences

Fay W. Boozman College of Public Health

University of Arkansas for Medical Sciences

4301 W Markham St., #820

Little Rock, AR 72205-7190

Email: PHsu@uams.edu

Office: 501-526-6687

**Supplemental Table 1**. Adjusted^a^ multivariable logistic regression associations between self-reported smoking status^b^ and high-aggressive prostate cancer^c^ stratified by self-reported race.

|  | Smoking Status | High-/Low-aggressive cases | OR | 95% CI |
| --- | --- | --- | --- | --- |
| Main effect | Never | 88/365 | 1.00 | (ref.) |
|  | Former > 23 years | 79/222 | 1.36 | 0.94-1.96 |
|  | Former < 23 years | 91/239 | 1.30 | 0.91-1.86 |
|  | Current | 72/123 | 1.99 | 1.29-3.05 |
| Self-reported race |  |  |  |  |
| African American | Never | 34/155 | 1.00 | (ref.) |
|  | Former > 23 years | 36/72 | 2.31 | 1.30-4.09 |
|  | Former < 23 years | 59/115 | 2.16 | 1.30-3.61 |
|  | Current | 58/75 | 3.41 | 1.95-5.98 |
| European American | Never | 54/210 | 1.00 | (ref.) |
|  | Former > 23 years | 43/150 | 0.94 | 0.59-1.51 |
|  | Former < 23 years | 32/124 | 0.86 | 0.52-1.44 |
|  | Current | 14/48 | 0.98 | 0.48-1.99 |
|  | *P* (multiplicative interaction by race) = 0.01 | |  |  |

^a^ Adjusted for age (in age-unstratified model), self-reported race (in race-unstratified model), family history, screening history, body mass index, health insurance, education, and income.

^b^ Smoking status defined by survey questions on whether participants had smoked more than 100 cigarettes (5 packs) in their lifetime, and if they were currently smoking at time of prostate cancer diagnosis. Current (>100 cigarettes and currently smoking), former (>100 cigarettes and not currently smoking), never (<100 cigarettes). Former smokers were further dichotomized by the median years since cessation value among low-aggressive cases.

^c^ Prostate cancer aggressiveness defined by Gleason sum, tumor stage, and PSA level at diagnosis. High-aggressive (Gleason sum ≥8 or PSA >20 ng/mL or Gleason sum = 7 and tumor stage 3-4), low-aggressive (Gleason sum < 7 and tumor stage 1-2 and PSA <10 ng/mL).

**Supplemental Table 2**. Adjusted^a^ multivariable logistic regression associations between self-reported smoking status^b^ and high-aggressive prostate cancer^c^ stratified by self-reported race.

|  | Smoking Status | High-/Low-aggressive cases | OR | 95% CI |
| --- | --- | --- | --- | --- |
| Main effect | Never | 88/365 | 1.00 | (ref.) |
|  | Tertile 1 | 72/195 | 1.35 | 0.93-1.97 |
|  | Tertile 2 | 87/195 | 1.58 | 1.10-2.28 |
|  | Tertile 3 | 83/194 | 1.42 | 0.97-2.07 |
| Self-reported race |  |  |  |  |
| African American | Never | 34/155 | 1.00 | (ref.) |
|  | Tertile 1 | 53/99 | 2.34 | 1.39-3.95 |
|  | Tertile 2 | 60/94 | 2.76 | 1.64-4.65 |
|  | Tertile 3 | 40/69 | 2.45 | 1.38-4.34 |
| European American | Never | 54/210 | 1.00 | (ref.) |
|  | Tertile 1 | 19/96 | 0.74 | 0.41-1.34 |
|  | Tertile 2 | 27/101 | 0.95 | 0.56-1.63 |
|  | Tertile 3 | 43/125 | 1.03 | 0.63-1.67 |
|  | *P* (multiplicative interaction by race) = 0.009 | |  |  |

^a^ Adjusted for age (in age-unstratified model), self-reported race (in race-unstratified model), family history, screening history, body mass index, health insurance, education, and income.

^b^ Smoking status defined by never vs. ever-smokers, with ever-smokers categorized into pack-year tertiles based on the low-aggressive ever-smoker distribution.

^c^ Prostate cancer aggressiveness defined by Gleason sum, tumor stage, and PSA level at diagnosis. High-aggressive (Gleason sum ≥8 or PSA >20 ng/mL or Gleason sum = 7 and tumor stage 3-4), low-aggressive (Gleason sum < 7 and tumor stage 1-2 and PSA <10 ng/mL).
